# Genomic sequence and activity of KS10, a transposable phage of the *Burkholderia cepacia complex*

**DOI:** 10.1186/1471-2164-9-615

**Published:** 2008-12-18

**Authors:** Amanda D Goudie, Karlene H Lynch, Kimberley D Seed, Paul Stothard, Savita Shrivastava, David S Wishart, Jonathan J Dennis

**Affiliations:** 1 Department of Biological Sciences, University of Alberta, Edmonton, Alberta, Canada; 2 Department of Agricultural, Food and Nutritional Science, University of Alberta, Edmonton, Alberta, Canada; 3 Department of Computing Science, University of Alberta, Edmonton, Alberta, Canada

## Abstract

**Background:**

The *Burkholderia cepacia *complex (BCC) is a versatile group of Gram negative organisms that can be found throughout the environment in sources such as soil, water, and plants. While BCC bacteria can be involved in beneficial interactions with plants, they are also considered opportunistic pathogens, specifically in patients with cystic fibrosis and chronic granulomatous disease. These organisms also exhibit resistance to many antibiotics, making conventional treatment often unsuccessful. KS10 was isolated as a prophage of *B. cenocepacia *K56-2, a clinically relevant strain of the BCC. Our objective was to sequence the genome of this phage and also determine if this prophage encoded any virulence determinants.

**Results:**

KS10 is a 37,635 base pairs (bp) transposable phage of the opportunistic pathogen *Burkholderia cenocepacia*. Genome sequence analysis and annotation of this phage reveals that KS10 shows the closest sequence homology to Mu and BcepMu. KS10 was found to be a prophage in three different strains of *B. cenocepacia*, including strains K56-2, J2315, and C5424, and seven tested clinical isolates of *B. cenocepacia*, but no other BCC species. A survey of 23 strains and 20 clinical isolates of the BCC revealed that KS10 is able to form plaques on lawns of *B. ambifaria *LMG 19467, *B. cenocepacia *PC184, and *B. stabilis *LMG 18870.

**Conclusion:**

KS10 is a novel phage with a genomic organization that differs from most phages in that its capsid genes are not aligned into one module but rather separated by approximately 11 kb, giving evidence of one or more prior genetic rearrangements. There were no potential virulence factors identified in KS10, though many hypothetical proteins were identified with no known function.

## Background

The *Burkholderia cepacia *complex (BCC) is a group of Gram negative, motile bacilli, first described in 1950 by W.H. Burkholder as the causative agent of soft rot in onion [1]. BCC species can be found throughout the environment, and have been isolated from sources such as soil, water, and plants [2]. BCC organisms are extremely diverse and versatile in their metabolic capabilities and although discovered as a plant pathogen, some strains of the BCC are actually beneficial to plants and to the environment. The BCC has also become known as an important group of opportunistic pathogens in immunocompromised patients, specifically in those with cystic fibrosis (CF) or chronic granulomatous disease (CGD) [3,4]. Since most clinically relevant strains of the BCC are resistant to multiple antibiotics, the most effective treatments against BCC bacteria involve specific antibiotic combinations. However, even with repeated combination antibiotic therapy in CF patients, clearance of the microorganisms is not observed. As an alternative treatment, bacteriophage (or phage) therapy is currently being researched and tested for the treatment of BCC infections [5].

Phage therapy, developed Felix d'Herelle, involves the use of lytic phages to kill infecting bacteria [6]. However, modified temperate phages or phage products may also be considered [7]. Phage therapy may prove to be more effective and efficient than antibiotics, especially since it is much easier to modify a phage than it is to develop a new antibiotic when bacterial strains become resistant. However, there are still a number of problems with using phages as therapeutic agents that must be overcome, one of these problems being lysogenic conversion. In order for phage therapy to be used safely, especially when involving temperate phages, it is important to determine if phages encode bacterial toxins or genes that could be harmful if acquired by the host bacterium. A Mu-like phage of the BCC, BcepMu, for example, encodes a putative ExeA homolog, which may be involved in the secretion of toxins, and a 3-O-acyltransferase homolog, which may be responsible for a bacterium's resistance towards certain antibiotics [8]. Other well-known examples of phages conferring virulence to their bacterial hosts are the cholera toxin of *Vibrio cholera*, the Shiga toxin of *E. coli*, and the scarlet fever toxin of *Streptococcus pyogenes *[9-11]. Therefore, phages should be sequenced to ensure that they do not harbor potential virulence determinants before they are used in a therapeutic setting.

In 2005, Seed and Dennis isolated four lytic phages from onion rhizosphere as well as five temperate phages from five different strains within the BCC [5]. One of these temperate phages, KS10, was isolated as prophage of *B. cenocepacia *strain K56-2. Most *B. cenocepacia *of ET12 lineage similar to K56-2, such as strain J2315, harbour a Mu-like prophage called BcepMu, while K56-2 does not [8]. A search of the previously determined *B. cenocepacia *J2315 genomic sequence, as well as a more extensive search for lysogeny in BCC strains, determined BcepMu to be the only known prophage of J2315 [12]. This study reports the host range, genome sequence and organization, and putative gene functions of the transposable phage KS10 in comparison to BcepMu and Mu. Analysis of the KS10 genome will allow us to identify any potential virulence determinants encoded by this temperate BCC phage.

## Results and Discussion

### Properties of lysogenic phage KS10

KS10 was originally identified as pinpoint plaques present on lawns of uninduced cultures of *B. cenocepacia *K56-2. In contrast to phage Mu, and many other Mu-like phages, KS10 in K56-2 appears to spontaneously switch to its lytic life cycle at a high frequency. Because of this, it was not necessary to use mitomycin C or exposure to UV light to initiate induction. The phage lysate from *B. cenocepacia *K56-2 plated on another BCC strain well-known to be susceptible to phage infection [5], *B. ambifaria *LMG 19467, produces slightly larger plaques than the small pinpoint plaques found on K56-2. To confirm that KS10 is a prophage of K56-2, KS10 was propagated on LMG 19467 and did not form plaques on K56-2, indicating resistance to superinfection. KS10 particles are structurally stable over a long period of time, with titers remaining as high as 6.75 × 10^6 ^pfu/ml after storage in suspension media (SM) for 10 months. Because of this trait, just one plaque of KS10 in 1 mL of SM can produce confluent lysis when plated with strain LMG 19467.

**Figure 1:**
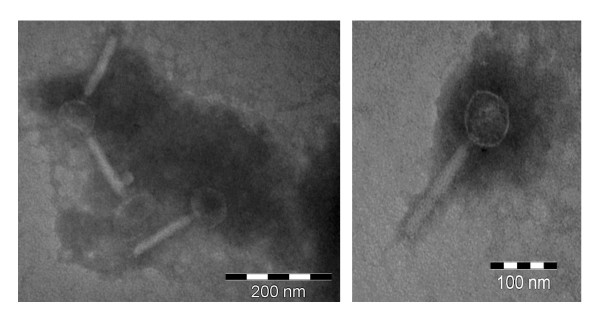
**Electron micrographs showing morphology of KS10 particles in lysate stored at 4°C for approximately one week prior to imaging**. Lysate was negatively stained with 2% phosphotungstic acid. Images were viewed at 110,000-fold magnification (A), and 140,000-fold magnification (B) using a Philips/FEI (Morgagni) Transmission Electron Microscope with CCD camera in the Biological Science Department's Microscopy Unit.

An electron micrograph (EM) of KS10 virions negatively stained with 2% phosphotungstic acid shows an icosahedral head and tail that appears to be contractile upon visualization (Figure 1). This is typical of the *Myoviridae *family of phages. EM analysis also reveals the average KS10 head size to be approximately 80 nm with a tail length of approximately 140 nm.

### Determination of the genome sequence

The KS10 DNA sequence was determined using a shotgun cloning and sequencing approach. Phage DNA was digested with restriction enzymes, ligated into pUC19 or pGEM7Z, and transformed into chemically competent DH5α cells. Inserts larger than 10 kb were also subcloned and sequenced. All inserts were sequenced at least twice, and PCR or primer walking was used to fill gaps in the sequence. Initial restriction digests suggested an estimated genome size of 35 kb. Approximately 249 runs with an average read-length of 680 bp were assembled to give greater then 4-fold genome coverage, resulting in a single contig totaling 37,635 bp in length. This DNA sequence had approximately 63% GC content, which is significantly higher than that of phage Mu, but similar to BcepMu. In order to determine if the recently completed *B. cenocepacia *J2315 genome was lysogenized by KS10, a BLASTX analysis was carried out at the Wellcome Trust Sanger Institute site , revealing that KS10 is indeed a prophage of *B. cenocepacia *J2315. A comparison of the KS10 sequence with the J2315 genomic sequence showed an exact KS10 sequence is located in chromosome 1 of J2315 (bp 1,766,551 to bp 1,728,918), on the complementary strand. As *B. cenocepacia *J2315 also harbours prophage BcepMu, this strain contains two functionally similar Mu-like phages, whereas *B. cenocepacia *K56-2 only contains the Mu-like phage KS10.

The right end phage insertion site, which varies across and within *B. cenocepacia *strains, was determined by sequencing two clones and one subclone from *B. cenocepacia *K56-2 that contained the phage/host DNA junction at two different insertion sites. These results indicated that there was random insertion occurring within the *B. cenocepacia *K56-2 genome, a feature characteristic of transposable phages. The left end phage insertion site was obtained using PCR of genomic DNA since no shotgun clones containing a phage/host insert were originally isolated. Forward primers were designed to the chromosome for the region upstream of the gene interrupted by the KS10 phage as determined for the right end insertion site, and reverse primers were designed to the left end of completed KS10 phage sequence.

### Host range and presence of KS10 within the BCC

Using a plaque assay, 23 BCC strains were tested for their sensitivity to KS10. Only three of the tested strains were found to support plaque formation with KS10: *B. ambifaria *LMG 19467, *B. cenocepacia *PC184, and *B. stabilis *LMG 18870. Lysates obtained from filter sterilized uninduced overnight cultures of both K56-2 and J2315 were able to form plaques on LMG 19467, PC184, and LMG 18870. Since *B. ambifaria *LMG 19467 (but not *B. cenocepacia *PC184) is also a host for BcepMu, lysate obtained from an overnight culture of *B. cenocepacia *J2315 will also form BcepMu plaques on a lawn of *B. ambifaria *LMG 19467. On these plates we observed a noticeable increase in the number of plaques, suggesting that both KS10 and BcepMu were being shed by J2315. It is possible that KS10 was not detected in previous attempts to isolate prophages from strain J2315 by assaying an overnight culture for PFU [12] due to its relatively poor host range, and its overlap in host range with BcepMu. Interestingly, although the lysogenic phase phage repressors of KS10 and BcepMu show 36% identity to each other, and both prophages are found integrated into *B. cenocepacia *strains such as J2315, in *B. cenocepacia *K56-2 only KS10 can exist as a prophage while BcepMu will form plaques on this strain.

**Figure 2:**
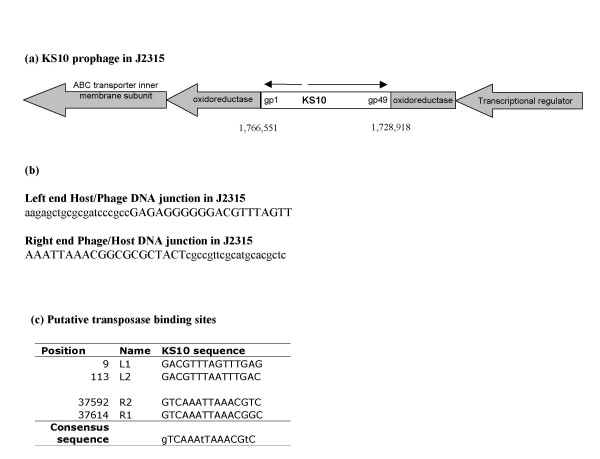
**Phage/Host DNA junction and putative transposase binding sites**. (A) KS10 has integrated into an oxidoreductase gene of *B. cenocepacia *J2315. Within the J2315 genome, bases 1,728,819 through 1,766,551 are KS10 prophage sequence. Direction of transcription of surrounding genes is indicated with thick arrows, while the direction of transcription of KS10 genes is indicated with thin arrows. The diagram is not drawn to scale. (B) Uppercase letters indicate KS10 sequence while lowercase represent *B. cenocepacia *J2315 host DNA. Phage right end is on the left in this diagram due to its orientation in the J2315 genome, while the phage left end is on the right. (C) Four putative transposase binding sites at the terminal ends of KS10. The position and sequence of the four 15 nucleotide direct repeats that are predicted to be TnpA binding sites in KS10 are indicated. The L1 and L2 transposase binding sites, at the left end, are inverted relative to the R1 and R1 binding sites at the right end. All sequences are written 5'-3'. Consensus sequence is written in the direction of the R1 and R2 binding sites.

In host K56-2, at the time of initial KS10 sequencing, the majority of the cloned host/phage sequences show that KS10 was inserted before amino acid 394 of a transcriptional regulator in the GntR family (Table 1). Since KS10 is a transposable phage, after transposition it can be inserted in many different locations within the genome. BLASTN analysis of the host DNA flanking the phage sequence from the Sanger site indicates that in *B. cenocepacia *J2315 the prophage is located in chromosome 1, and has inserted in an oxidoreductase gene in the Gfo/Idh/MocA family around amino acid 235. It has been suggested that prophages are arranged so that their structural genes are oriented in the same direction as the genes surrounding them, but the orientation of the KS10 prophage in J2315 orients the transcription of structural genes from right to left, while the surrounding genes are transcribed from left to right (Figure 2) [13,14].

**Table 1:** BCC strains/isolates testing positive for KS10 prophage.

Host (*B. cenocepacia *unless noted otherwise)	Source	Integration Site
K56-2	BCC experimental strain panel	Transcriptional regulator; GntR family with aminotransferase
J2315*	BCC experimental strain panel	Oxidoreductase on Chromosome 1
C5424*	BCC experimental strain panel	ND

R1882	Clinical Isolate	ND
R1883	Clinical Isolate	ND
R1884	Clinical Isolate	ND
S11528	Clinical Isolate	ND
R1434	Clinical Isolate	ND
R750	Clinical Isolate	ND
R2314	Clinical Isolate	ND

*B. ambifaria *LMG 19467 (lys1)	BCC experimental strain panel (modified, this study)	ND
*B. ambifaria *LMG 19467 (lys2)	BCC experimental strain panel (modified, this study)	ND
PC184 (lys3)	BCC experimental strain panel (modified, this study)	ND

* – Also known to contain BcepMu

ND – Not determined

To determine the distribution of KS10 within the genome of other BCC species/strains, PCR using primers specific to the KS10 sequence was used. *B. cenocepacia *K56-2 and J2315 chromosomes were used as positive controls since these strains are known to be lysogenized by KS10. *B. ambifaria *LMG 19467 genomic DNA was used as a negative control due to the ability of KS10 to form plaques on this strain. Seven strains of *B. cenocepacia *and one strain from each of the other species in the BCC experimental strain panel were tested [15,16]. Only one other BCC strain was found to be lysogenized with KS10, *B. cenocepacia *strain C5424, and this strain has been shown previously to contain an integrated BcepMu [8]. In addition, 27 BCC clinical isolates obtained from the University of Alberta Hospitals (Pediatric/Adult) Cystic Fibrosis Clinic were also tested, revealing that KS10 is a prophage in seven of these isolates. All seven isolates were characterized as *B. cenocepacia *based on *fur *gene sequence analysis, suggesting that KS10 is a temperate phage specific to *B. cenocepacia *of the BCC (Table 1) [17].

### Integration of KS10

We attempted to identify the exact location of KS10 in all known BCC hosts by locating the actual phage integration site. Arbitrary primers (ARB6 and ARB2) and a specific KS10 primer, in addition to the APA gene Gold genome walker kit (Bio S&T, Montreal), were used in PCR experiments to determine the precise insertion site of the integrated prophage. Unfortunately, PCR products obtained were found by sequence analysis to be the result of mispriming. This finding limited our ability to identify an exact site of insertion for KS10 in each *B. cenocepacia *genome, even though PCR tests unambiguously identified its presence in the genomes of these strains (Table 2).

**Table 2:** Phage KS10 putative genes and homologues

**GP**	**Coding**	**Str.**	Possible RBS* & Start Codon	**AA**	**Putative Function**	**Alignment Region**	**% Identity**	**Score**	**% GC**	**Sig. matches to proteins in NCBI's GenBank**
1	205–1437	-	AGGCGcgtaaATG	410	virion morphogenesis	400(28–418)/248(16–263)	30%/33%	160/137	63.26	Pseudomonas bacteriophage B3 gp33/BcepMu gp30
2	1438–1698	-	AAAGGAGccaacaATG	86	hyp. protein	-	-	-	61.69	no sig. match
3	1743–3278	-	GGAAG//36 bp//cgtttATG	511	portal	354(35–374)	32%	182	62.57	BcepMu gp29
4	3268–4884	-	AAGAGGcctgatccacgATG	538	terminase large subunit**	520(4–521)	55%	569	58.94	D3112 p26
5	4891–5388	-	AAGGGttgacgcATG	165	terminase small subunit**	164(1–164)	37%	161	59.24	D3112 p24
6	5392–5682	-	AGGGTATccgcgATG	96	hyp. protein	-	-	-	59.45	no sig. match
7	5679–5903	-	GGATAacgATG	74	dksA/traR C4-type zinc finger	74(1–72)	45%	44.3	60.89	Enterobateria phage WPhi gp82
8	5893–6186	-	GAGGAtgcccgtcATG	102	hyp. protein	-	-	-	65.99	no sig. match
9	6466–7137	-	GTGGGcctcgcagcATG	223	endolysin	186(20–203)	54%	188	65.03	BcepMu gp22
10	7134–7535	-	GGGTGccgccgtcgcgaATG	133	holin	119(1–111)/116(8–113)	36%/39%	72.4/63.5	58.96	*Ralstonia solanacearum *UW551***/BcepMu gp21
11	7650–8258	-	GGGAAGcgcgaaATG	202	hyp. protein	177(53–211)	26%	45.4	53.53	*Pseudomonas entomophila *L48
12	8251–8784	-	GGGAATAtgtaaagcATG	177	hyp. protein	-	-	-	54.87	no sig. match
13	8839–9309	-	AAGAGGccgaccATG	156	hyp. protein/phage repressor	131(1–125)/137(2–127)	39%/36%	85.1/72.4	56.69	*E. coli *B171/BcepMu gp17
14	9391–9582	+	GGAGcaaatATG	63	DNA binding protein/conserved hyp. protein	62(5–65)/59(1–57)	33%/52%	38.1/65.1	58.85	BcepMu gp16/*Ralstonia solanacearum *UW551
15	9579–10631	+	GGGGAGGtggATG	350	conserved hyp. protein	141(49–186)/127(77–203)	28%/28%	38.1/44.7	64.71	BcepMu gp10/*Ralstonia solanacearum *UW551
16	10671–12296	+	AGGTGcgatATG	541	transposase	539(24–559)	36%	336	65.19	*Ralstonia solanacearum *UW551
17	12306–13298	+	GAATAAGGAGtgaccATG	330	transposition protein	309(7–311)	42%	222	64.15	*Ralstonia solanacearum *UW551
18	13306–13494	+	GTGAGGccatcATG	62	hyp. protein	-	-	-	66.14	no sig. match
19	13491–13796	+	GAGGTGAcggcATG	101	hyp. protein	-	-	-	69.28	no sig. match
20	13859–14371	+	TGGccgacgacagcATG	170	hyp. protein	172(55–187)	60%	180	67.25	*Ralstonia solanacearum *UW551
21	14368–14994	+	AAGGAAcccatcATG	208	conserved protein	200(9–210)/199(6–204)	72%/55%	210/205	63	*Ralstonia solanacearum *UW551/BcepMu gp05
22	15005–15397	+	GAGGGGctggccATG	130	hyp. protein	-	-	-	66.16	no sig. match
23	15460–15732	+	AGGAGAAAcaccctcATG	90	DNA binding protein Hu-beta	90(1–90)	57%	108	64.47	*Bordetella pertussis *Tohama I
24	15809–16642	+	GAGGAAccccaaaATG	277	hyp. protein	117(51167)/58(344–401)	53%/67%	114	62.83	*Burkholderia thailandensis *E264 gp38
25	16806–17126	+	GGGGAGTGAcactgtgATG	106	hyp. protein	111(9–119)	37%	55.1	64.49	*Burkholderia pseudomallei *K96243
26	17128–17568	+	GGAGGcccgctgacATG	146	modulation of host genes?	129(9–135)	30%	58.5	64.17	*Escherichia coli *B7A/Mu gp16
27	17565–17963	+	GAAACGAccgcATG	132	middle operon regulator (Mor)****	106(14–118)	31%	52.8	64.91	*Pseudomonas entomophila *L48
28	18120–19322	+	AGAGAAccatTTG	400	protease	330(10–322)	33%	147	65.59	BcepMu gp32
29	18876–19322	+	AGGAagccATG	148	scaffold	-	-	-	67.11	BcepMu gp33
30	19368–19724	+	AGAGGATtcacATG	118	conserved hyp. protein	77(41–117)/80(32–109)	54%/42%	72.4/44.3	71.71	Bacteriophage B3 gp37/BcepMu gp35
31	19775–20722	+	GGAGctatccATG	315	major head subunit	311(4–307)	47%	243	64.87	Bacteriophage B3 gp34
32	20797–21186	+	AAGAGAGatcATG	129	hyp. protein	-	-	-	66.92	no sig. match
33	21183–21686	+	GAAGGGccgcaaATG	167	hyp. protein	-	-	-	65.48	no sig. match
34	21683–22114	+	GGATAGGTAcgggaaATG	143	virion morph.	161(8–156) 121(7–124)	31%/36%	161/52.4	60.19	*Thermus aquaticus *Y51MC23/*Escherichia coli *53638 (MuG)
35	22114–22716	+	GGTGAGGATGAtgtgATG	200	phage-related conserved hyp. protein	165(1–164)	24%	41.2	61.36	*Burkholderia thailandensis *MSMB43
36	22700–22978	+	ACGGG//25 bp//gatATG	92	hyp. protein	-	-	-	64.52	no sig. match
37	23022–24500	+	AAGGGAcattcgacATG	492	tail sheath protein	472(12–481)	34%	188	63.15	Prophage MuSo2 (*Marinomonas *sp.)/MuL (gp39)
38	24546–24917	+	AAGGGAGTGaaacATG	123	hyp. protein	-	-	-	62.9	no sig. match
39	25000–25554	+	TGAGATTcccaccATG	184	tail assembly chaperone	-	-	-	62.88	FluMu gp41**
40	25603–28038	+	GAGGAAGAgacgATG	811	tail tape measure (TP109 fam.)	484(3–458)	38%	265	64.2	*Burkholderia vietnamiensis *G4
41	28038–29408	+	GGAGGAAcgaactgATG	456	DNA circulation protein	455(3–412)	27%	150	65.43	*Polaromonas *sp. JS666/MuN (gp47)
42	29414–30571	+	TGAcccctATG	385	tail protein	352(96–337)	33%	153	63.04	*Polaromonas *sp. JS666/MuP (gp44)
43	30571–31092	+	GGAGcaaactgATG	173	baseplate assembly	60(36–188)	30%	63.5	61.88	*Escherichia. coli *0157:H7 str. EC4501/MuQ (gp45)
44	31177–31758	+	AGGccatcATG	193	tail protein	99(25–112)	47%	70.9	64.6	MuV (gp46)
45	31755–32876	+	GGAAAAcatcATG	476	tail protein	306(24–325)	36%	144	65.86	*Pseudomonas entomophila *L48/MuW (gp47)
46	32879–33478	+	AGGAGTgaccGTG	173	tail protein	197(5–192)	26%	57	65.17	*Desulfovibrio vulgaris *subsp. vulgaris str. Hildenborough
47	33478–34455	+	GGAcatcgactgATG	325	tail fiber protein	229(310–534)	42%	140	62.47	*Burkholderia multivorans *ATCC 17616
48	34463–36691	+	TGAGGcacgcATG	742	ABC-type phosphate transport sys.	459(40–493)	46%	364	60	*Pseudomonas stutzeri *A1501
49	36711–37475	+	GAGGTAcaaATG	254	hyp. protein	270(1–248)	28%	81.3	59.48	Klebsiella pneumoniae 342 KPK_4114

Abbreviations: GP, gene product; Str., strand; RBS, ribosome-binding site; AA, amino acid; hyp, hypothetical; sig, significant; subsp, subspecies;

*Putative RBS binding sites for gp1, gp4, and gp37 were identified using RBS finder.

**Function predicted using PSI-BLAST

***All *R. solanacearum *UW551 references refer to unfinished DNA segment NZ_AAKL01000024.1

****Function predicted using PHYRE database

Normally, KS10 will form plaques on lawns of *B. ambifaria *LMG 19467 and *B. cenocepacia *PC184, but at a lower frequency KS10 will also enter into its lysogenic life cycle. To demonstrate that KS10 can insert into the chromosomes of these hosts, colonies of strains that had been lysogenized with KS10 were selected. In order to obtain these lysogens, KS10 was plated with LMG 19467 and PC184 and turbid plaques indicative of lysogeny were selected using a needle tip. Two LMG 19467 lysogens and one PC184 lysogen were collected and demonstrated by PCR to harbour KS10.

### KS10 potential genes and homologues

GeneMark and NCBI's ORF Finder programs were used to identify open reading frames within the KS10 genome sequence [18]. Each identified ORF was characterized using BLAST analysis against deposited sequences in the NCBI databases in order to assign a possible function (Table 2). Regions upstream of the ORFs were examined to determine potential ribosome binding sites (RBS) between 4 and 14 bases upstream of the start codon [19]. A total of forty-nine putative genes were identified, with just less than half encoding hypothetical proteins possessing no known function. KS10 genes 11 and 12 have significantly lower GC content than the genome overall (Table 2) suggesting that these genes were acquired by KS10 more recently. All putative genes utilize AUG as their translational start codon, except for two possessing a UUG and GUG start codon.

BLAST analysis (NCBI) showed that approximately 18% of KS10 proteins are homologous to proteins of BcepMu, whereas another 18% show homology to Mu proteins [20]. 14% of KS10 proteins show identity to proteins from *Ralstonia solanacearum *strain UW551. Although they are not annotated as such in the UW551 GenBank entry, these are likely proteins of a prophage and not bacterial proteins. The highest percent identity that KS10 protein sequences exhibit to an orthologous protein is 67%, while the majority of KS10 proteins show only moderate identity and similarity to other phage proteins (Table 2). Because DNA recombination can often occur between prophages in the same bacterial genome, it is perhaps surprising that none of the KS10 genes show higher homology to BcepMu genes, since KS10 was found to be a prophage in several *B. cenocepacia *strains that are also lysogenized with BcepMu.

The genes identified as encoding hypothetical proteins using the standard BLASTX searches were subjected to analysis with PSI-BLAST. This program uses the predicted amino acid sequences of each gene to detect putative conserved domains within the sequence [21]. Using this program, KS10 gp4 and gp5 were identified as being the large and small terminase subunits, which are predicted to be ATP-binding proteins involved in DNA packaging into the procapsid [22]. These putative terminase genes are physically located after the virion morphogenesis and portal genes, an order that is conserved in many phage genomes [8,22]. Similarly, gp39 was found to show homology to FluMu41 using PSI-BLAST, a protein that is thought to be analogous to Lambda G, a tail assembly chaperone [23].

Higher order bioinformatic analysis was performed using in-house programs for protein domain identification and functional prediction [24]. These analyses confirmed the results obtained using BLAST analyses but provided little additional functional protein information (see Additional file 1). Potential virulence factor genes were not identified within the genome of KS10. However, there remain several genes encoding hypothetical proteins within the KS10 genome that cannot be excluded as potential virulence factors until their functions are determined.

### Head assembly genes

In most phages, head assembly involves five major stages, each involving a number of phage encoded proteins. The first stage, initiation, involves the initiation of polymerization of phage coat proteins by minor head proteins. Next, during shell formation, the immature procapsid, comprised of coat proteins, is assembled around the scaffold protein, which is subsequently cleaved to form a mature procapsid. Following the maturation of the procapsid are the two final stages, DNA packaging and head completion [25]. In KS10, gp1-gp5 and gp28-gp34 are expected to be involved in head assembly based on homology. KS10 gp28 and gp29 are identified as the protease and scaffolding proteins, which are structurally important to this process. The scaffolding protein has been shown to be essential in assembling coat proteins, encoded by gene 31 in KS10, into the capsid shell [26]. Once this shell is formed, it is thought that the protease, encoded by KS10 gene 28, is involved in removing this scaffold. As is the case in many other phages, the scaffold gene of KS10 is embedded in frame with the protease gene.

The proteins involved in the actual packaging of the DNA into the procapsid are the terminases, portal, and major capsid, which in KS10 are identified as gp4, gp5, gp3, and gp31 respectively. KS10 gene 34 encodes a protein that shows homology to MuG, which is thought to be a tail protein, though its function is unclear [27]. This gene also shows no similarity to other known genes in other non-Mu-like phages [22]. KS10 gene 1 encodes a protein putatively involved in virion morphogenesis since it exhibits identity to MuF, though its function remains unknown. There are also a number of genes encoding hypothetical proteins interspersed within these genes, which could encode minor proteins involved in forming the mature capsid.

### Host Cell Lysis

Phage host cell lysis usually involves a number of proteins, including a holin and a lysin. In KS10, these proteins are encoded by gene 10 and gene 9, respectively. There are a variety of lysins that can be phage encoded, all of which are peptidoglycan hydrolases. Holin proteins are hydrophobic and associate with the cytoplasmic membrane of the bacteria, creating holes that allow the lysin to move into the periplasm. KS10 holin and lysin proteins show homology to the holin and lysin of BcepMu, but the lysins differ in the N-terminal region [8]. PSI-BLAST analysis revealed the lysin of KS10 to have a soluble lytic transglycosylase (Slt) domain of 117 amino acids, similar to that of BcepMu and other non-Mu phages such as T7. Most enzymes of this nature found in *E. coli *have been shown to catalyze the cleavage between N-acetylmuramic acid and N-acetylglucosamine, resulting in 1,6-anhydromuramic acid. In *E. coli*, these enzymes degrade the cell wall murein during bacterial morphogenesis [28]. In BcepMu this Slt domain is conserved over residues 46–155, while in KS10 the domain is conserved over residues 70–170. Most phage lysins lack their own signal peptide (SP) sequence, and are under control of the holin for release. In a search for encoded SP sequences within the lysin gene of KS10, however, a potential SP sequence was identified using SignalP server . The cleavage site in KS10 gp10 was predicted to be between amino acids 44 and 45. The N-terminal region of KS10 putative lysin protein is also fairly hydrophobic, further suggesting that this protein contains an SP, although this activity has not been confirmed experimentally. Although not a common phenomenon, lysins of other phages, such as the *Oenococcus oeni *phage fOg44, have been found to contain an SP sequence. In this phage, overexpresssion of the lysin (Lys44) in *E. coli *was lethal, which is uncommon for phage lytic enzymes. When the SP was deleted, overexpression of Lys44 was no longer toxic [29]. Other phage genes that encode secondary lysis proteins such as Rz/Rz1 were not identified in KS10.

### Transposition

The proteins involved in transposition of KS10, specifically those encoded by gene 16 and gene 17, show the most homology to a transposase and transposition protein found in the genome of *R. solanacearum *strain UW551. The KS10 transposase shows little homology to BcepMu and other Mu-like transposases that are closely related to the *S. auereus *transposon Tn*552 *[8]. The integrated KS10 lacks the 5'-TG...CA-3' dinucleotides at its termini, which are characteristic of Mu-like prophage elements related to BcepMu carrying an Rve integrase catalytic core domain [8]. However, the KS10 transposase does exhibit 30% identity and 44% similarity with the transposase from the transposable *Pseudomonas *phage D3112 [30]. Interestingly, where the KS10 prophage is integrated, no evidence of direct repeats has been identified in the flanking BCC DNA. The putative transposase binding sites for KS10 are imperfect direct repeats of 15 nucleotides. They do not appear to be similar to those found in BcepMu or *E. coli *Mu, which correlates well with the transposase of KS10 not showing significant homology to transposase of Mu or BcepMu. However, the putative KS10 transposase binding sites do contain the characteristic repeated A nucleotides within its target sequence (Figure 2). Unlike the prophages Mu, FluMu, Pnm1, and Sp18, which have 6 putative transposase binding sites within their genomes, there are only 4 identified in KS10 [18]. These putative sites in KS10 were identified after a search for the consensus TnpA binding sites in both Mu and BcepMu were not identified in the KS10 sequence (Figure 2).

Gyrase binding sites have been identified in Mu and some other Mu-like phages, and are thought to promote the replicative transposition process that occurs during Mu lytic growth. In Mu and FluMu this binding site is located between Mu gpG and Mu gpI [18]. A search for this site in KS10 revealed no similar sequence, though we predict that this binding site is present, as a previous study by Sokolsky and Baker revealed that gyrase is necessary in Mu replicative transposition [31]. By using a drug that inhibits gyrase, they concluded that gyrase activity is important for the lytic life cycle of phage Mu.

### Tail Assembly

Unlike proteins involved in head assembly, lysis, and transposition, there is little known about the proteins involved in Mu tail assembly. The last approximately 14 kb of the KS10 genome is involved in tail assembly. Like other members of the *Myoviridae *family, Mu-like phages have contractile tails, made up of a contractile sheath outside of an inner tail tube. The phage baseplate is located at the end of the tail and is attached to the tail fibers, which are involved in attachment to the host cell. KS10 genes 37 and 41 encode the tail sheath and tail tape measure proteins, respectively. In both Mu and BcepMu, the tail tube gene is found between the genes encoding the tail sheath and the tape measure. In KS10 there is a hypothetical protein of 123 amino acids that, using a BLAST analysis, shows no homology or conserved domains to any protein in the database. This gene is the correct length and in the expected location to encode a tail tube protein based on other similar phages, but there is no experimental evidence to support this claim.

Xu et al. suggest that there is a -1 (or -2 in Mu) frameshift conserved amongst dsDNA tailed phages that occurs before the tail tape measure gene and after the major tail gene [32]. This frameshift occurs in many phages including Mu, FluMu, P2, lambda, and D3. We found no evidence of a frameshift region before the tape measure gene in KS10. Using PSI-BLAST analysis, a FluM-like gp41 conserved domain was detected, which has been annotated as a Lambda G analogue. In many dsDNA tailed phages there is a "slippery" sequence within this gene that causes a frameshift creating two overlapping ORFs. This sequence is usually a region of repeated A, T, or G nucleotides. In KS10, a sequence capable of causing this frameshift was not identified using both a manual search as well as the Frame Shift Finder program .

KS10 gp42 to gp45 are predicted to be involved in baseplate assembly since they show homology to Mu proteins with this function. How each protein is involved in baseplate assembly in Mu is yet unknown. KS10 gene 47 encodes a 325 amino acid protein showing 42% identity to a tail collar protein of a *B. multivorans *strain prophage and 27% identity to a tail fiber protein of a prophage in *B. thailandensis *strain E264. In many Mu-like phages, the gene or genes encoding tail fibers is/are relatively long. BcepMu, for example, has a tail fiber gene encoding a 786 amino acid protein, similar to the phage P2, which is much larger than the small gene in KS10 showing low identity to a tail fiber. KS10 gp48, a 742 amino acid protein at the end of the genome shows 46% similarity to the periplasmic component of an ABC-type phosphate transporter system of *Pseudomonas stutzeri *A1501. However, the region showing homology to the gene from *P. stutzeri *shows no conserved domains when analyzed using PSI-BLAST and no homology to any other periplasmic component from an ABC- type phosphate transporter system, suggesting that this is probably not the function of this protein. Using the GTOP sequence homology search  to compare KS10 gp48 to all viruses in the database, the amino acid sequence of KS10 gp48 showed homology to other phage proteins annotated as being involved in host specificity and putative tail-host specificity. Though this homology was relatively low (25%), it suggests that this 742 amino acid protein is a tail fiber protein involved in recognizing the phage receptor on the host cell, and not part of an ABC-type phosphate transport system.

### Organization of the KS10 genome

**Figure 3:**
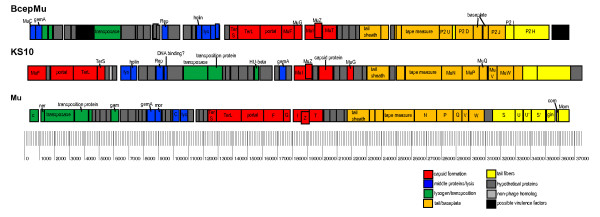
**Genome maps of KS10 and related phages BcepMu and Mu (derived from NC_005882 and NC_000929, respectively)**. Each box represents a predicted gene drawn to scale using GenVision program (DNASTAR) and arrows indicate direction of transcription. Homologues and known phage proteins are indicated (Table 1). Different colors represent different modules. Dark grey boxes indicate genes with no known phage homologues and are annotated as hypothetical proteins.

Unlike other sequenced phages similar to Mu, the first 5 gene products of KS10 are involved in head assembly. Following these genes in the KS10 genome are a number of genes involved in host cell lysis, followed by genes involved in transposition/integration, followed by additional genes involved in head assembly, and finally genes involved in tail formation. Mu-like phages, as is the case with most related phages, are usually genetic mosaics of each other and are often arranged in modules so that genes encoding proteins that interact, such as capsid genes, will not be separated by nonhomologous events [33]. The KS10 genome, however, has genes responsible for capsid formation separated, with virion morphogenesis, portal, and terminase genes at the beginning of the genome (Figure 3). This is uncommon for a Mu-like phage, as Mu phages generally have genes for head assembly in the middle of the genome, in the late gene region, which is usually more highly conserved than the early and middle gene regions [22]. To the best of our knowledge, this organization (dividing the capsid module) is unique to KS10. The other genes involved in head assembly, such as the protease, scaffold, and a MuG homologue, are located in the middle of the genome as expected.

**Figure 4:**
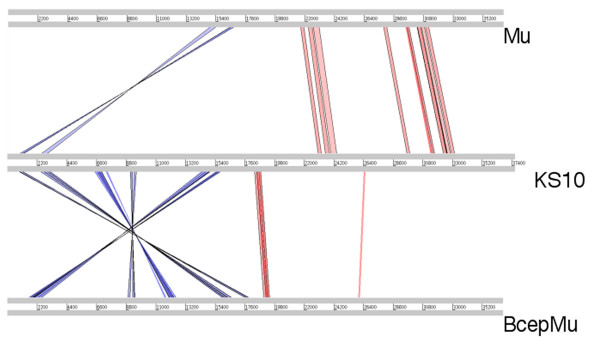
**Mu relationships identified using Artemis Comparison Tool program**. Translated BLAST (tblastx) was used to align translated genomic sequences of phages KS10, BcepMu, and Mu. An E-value cutoff of 10 and a score cutoff of 40 were used in this comparison. Nucleotide basepairs are indicated between grey lines for each phage genome. The blue and red lines represent the reverse and forward matches, respectively, and color intensity is proportional to the sequence homology.

When the KS10 genome is compared with the genome of Mu, the first approximately 17 kb of KS10 genome is the most varied, and appears to be inverted (Figure 4), with genes responsible for host cell lysis located between integration and head assembly genes. The organization and direction of transcription in KS10 allows the genes responsible for integration and transcription regulation to interrupt the head assembly module. It is unknown why this phage, found in multiple *B. cenocepacia *genomes, would have its genome arranged this way. Previous theories of phage evolution imply that evolution by illegitimate recombination usually occurs by recombination events that will not interrupt the individual modules, as is seen in the KS10 genome [33]. Since the protease and scaffold genes are found in the middle of the genome, it is unlikely that the head assembly proteins encoded earlier would be used until these genes have been transcribed and translated. The module encoding proteins for tail assembly is located at the right end of the genome, similar to Mu, though it lacks the invertible G region of Mu. This invertible region found in Mu and some related phages, encodes the proteins involved in tail fiber synthesis, and the orientation of the region determines the host range of the phage [34]. BcepMu, a similar phage found in *B. cenocepacia*, has a right end similar to P2 and also lacks this invertible G region [7]. Although KS10 tail protein gene sequences do show homology to Mu tail protein gene sequences, KS10 also lacks this invertible G region.

Often phages contain genes that they have acquired through nonhomologous recombination with a host or another phage. These acquired genes may encode proteins that can be involved in lysogenic conversion of the host cell, causing the bacterial host to become more virulent [33]. In order to test the hypothesis that KS10 contains genes whose products may increase the virulence of the bacterial host, we compared the killing effect of *B. ambifaria *LMG 19467 versus two strains of *B. ambifaria *19467 lysogenized with KS10 in the recently developed BCC *Galleria mellonella *infection model [35]. We also tested wild type and KS10 lysogenized *B. cenocepacia *PC184 in this model. In all instances, the virulence of the bacterial strain harbouring the KS10 prophage was similar to that of the bacterial strains without the prophage, suggesting that KS10 does not carry virulence factor genes that are expressed in vivo (data not shown).

A gene in KS10 that shows no homology to other known phage proteins is gene 7. KS10 gp7 shows relatively high identity to a dksA/traR C4-type zinc finger protein found in bacteriophage L-413C. DksA is a DnaK suppressor protein, which acts by suppressing transcription of DnaK, while TraR is a transcriptional activator. The coliphages P2, 186, and phage Phi MhaA1-PHL101 also encode a protein showing homology to this dskA/traR protein, though other phages similar to KS10 do not. In Phi MhaA1-PHL101 the conserved Dsk/TraR region is extends over the last 40 amino acids and it is hypothesized to be involved in transcriptional activation [36]. In KS10 gp7 the domain is conserved across the whole protein. This gene is located upstream of the first module of head genes. A possible role for this protein in KS10 could be to repress the transcription of genes 1–5 until the second head module is transcribed. If so, this protein may be responsible for controlling and coordinating the transcription of the two head gene modules.

While the KS10 genome seems to show unusual variability in its genomic organization, especially in the first half, it is possible that there are many other phages with similar organizations that have not yet been sequenced. Phages are one of the most abundant particles on Earth, but there are only a relatively small number of these phages whose genomes have been sequenced, making it difficult to draw definitive conclusions about the relationship of KS10 and its gene products to other phages. However, complete sequencing of bacterial genomes has provided increasing opportunities for prophage identification, and has also produced incontrovertible evidence of extensive phage-mediated exchange of genetic material between species. Although previous publications have suggested that Mu-like prophage elements are either rare or sufficiently divergent to be unrecognizable by sequence comparison, we have shown that polylysogeny does indeed occur in the BCC and that polylysogeny can occur with two different Mu-like BCC phages [8,12]. Further studies are required to understand the interactions of multiple active phages within a single genome of a strain of the BCC and their influence on cellular lifestyle and bacterial pathogenicity. The characterization of broad-host range BCC phages, regardless of whether they are lytic or lysogenic, will provide an opportunity to further develop these phages as novel therapeutic agents for use against infections caused by the highly antibiotic resistant BCC.

## Conclusion

KS10 is a novel 37,635 bp phage of the opportunistic bacterial pathogen *Burkholderia cenocepacia*. Genome sequence analysis and annotation of this phage reveals that KS10 shows the closest sequence homology to the transposable phages Mu and BcepMu. KS10 differs from most phages in that its capsid genes are arranged into two modules, giving evidence of one or more prior genetic rearrangements. KS10 was found to be a prophage in three different strains of *B. cenocepacia*, including strains K56-2, J2315, and C5424, and seven tested clinical isolates of *B. cenocepacia*, but no other BCC species. A survey of 23 strains and 20 clinical isolates of the BCC revealed that KS10 is able to form plaques on lawns of *B. ambifaria *LMG 19467, *B. cenocepacia *PC184, and *B. stabilis *LMG 18870. There were no potential virulence factors identified in KS10, though many hypothetical proteins were identified with no known function.

## Methods

### Bacterial strains, phages, and media

BCC strains including *B. cenocepacia *from the BCC experimental strain panels were obtained from the Belgian Coordinated Bacteria Collection M (Ghent, Belgium), or the Canadian *Burkholderia cepacia *complex Research and Referral Repository (Vancouver, Canada) [15,16]. BCC clinical isolates were obtained from the University of Alberta Hospitals Cystic Fibrosis Clinic (Edmonton, Canada). 1/2 concentration Luria Bertani (LB) broth or solid media was used to grow BCC host strains. LB solid media supplemented with ampicillin (0.1 g/L) was used to grow competent *E. coli *strains harbouring pUC19 or pGEM7Z. Growth of BCC strains was carried out aerobically 30°C overnight, while *E. coli *DH5α used for cloning was grown aerobically overnight at 37°C. KS10 plaques were picked from uninduced lawns of K56-2 and stored at 4°C in suspension media (SM) (50 mM Tris/HCl, pH 7.5, 100 mM NaCl, 10 mM MgS0_4_, and 0.01% gelatin solution). Phage stocks were prepared by placing an agar plug containing a single plaque into 1 mL of SM using a sterile glass Pasteur pipette and stored at 4°C.

### Bacteriophage production and host range testing

To determine the titer of one KS10 plaque in 1 ml of SM, the lysate was serially diluted in SM and plaque forming units (pfu) were determined using the soft agar overlay method. To determine host ranges, the KS10 phage stock was mixed with 23 different individual strains of BCC and 27 clinical isolates of BCC in soft agar overlays and plaques were tallied after 18–22 hours growth. KS10 distribution within the species of the BCC was determined using a PCR assay with oligonucleotide primers F3 (5'-CCGATTCCCACATCACGATCC) and R3 (5'-TGCGGGCATTTCAGCTTTCG). Bacterial genomic DNA was prepared as previously described [37]. PCR was performed in 50 μl reactions containing approximately 20 ng of template DNA and 25 pmol of each primer using *Taq*PCRx DNA Polymerase, Recombinant (Invitrogen) for one cycle at 96°C for one minute, 30 cycles at 96°C for 30 seconds, 70°C for 30 seconds, 72°C for 1 minute, and one cycle at 72°C for two minutes. To ensure the authenticity of the KS10 product, PCR products were analyzed by agarose gel electrophoresis and purified for sequencing using a QIAquick PCR purification kit (Qiagen Inc., Mississauga, Ont.).

### Transmission electron microscopy (TEM)

KS10 was obtained from an overnight culture of K56-2 (OD_600 _of approximately 2.0). Culture was centrifuged at 10,000 rcf for 2 minutes and filter sterilized using 0.45 μm filters. Filtrate was spotted onto copper grids and stained with 2% phosphotungstic acid. Micrographs were obtained using a Philips/FEI (Morgagni) Transmission Electron Microscope with CCD camera (Microscopy Unit, Biological Sciences, University of Alberta).

### Phage DNA Isolation and Sequencing

KS10 was propagated on host *B. ambifaria *LMG 19467 for DNA extraction. KS10 DNA was isolated from bacteriophage lysate using the Wizard Lambda DNA purification system (Promega Corp., Madison, WI). The purified KS10 DNA was digested using the restriction enzymes *Sph*I, *Eco*RI, and *Xho*I (Invitrogen Corp., Carlsbad, CA). To create cloned phage genomic DNA libraries, phage DNA fragments were purified using GeneClean II kit (QBiogene) and ligated into pUC19 or pGEM7Z. Ligated plasmids were transformed into chemically competent *E. coli *DH5α and the plasmids were purified for sequencing using a Qiaprep miniprep kit (Qiagen Inc., Mississauga, Ont.). Sequencing of the KS10 inserts was carried out using the ABI BigDye Terminator Cycle Sequencing Kit (Applied Biosystems) with an ABI 3100 Gene Analyzer (Molecular Biology Service Unit, Biological Sciences, University of Alberta). Sequencing data was edited using EditView 1.0.1 (Applied Biosystems) and assembled using AutoAssembler (Applied Biosystems). GeneMark and NCBI's ORF Finder programs were used to detect possible open reading frames (ORFs) [18,20]. Where multiple start codons or ORFs were indicated, the presence of a potential ribosomal binding site (RBS) was used to help identify the most likely ORF. Each identified ORF was tested with BLASTX analysis  to assign putative functions [20]. When BLASTX revealed no significant matches, PSI-BLAST (NCBI) was also used [21]. Comparisons of KS10 sequences with the *B. cenocepacia *J2315 genome sequence were carried out using the BLAST server at the Sanger Centre sequencing project web site ). Comparative maps were constructed using GenVision software (DNASTAR, Inc., Madison, Wis.) and Artemis Comparison Tool (Sanger Centre, UK) [38]. The complete annotated DNA sequence of bacteriophage KS10 can be found in GenBank under accession number EU822883.

### Characterization of PC184 and LMG 19467 lysogens

KS10 was propagated on BCC strains PC184 and LMG 19467 using the soft agar overlay method. Turbid plaques were identified, picked using a twenty-gauge needle, and placed in 1 mL of 1/2 LB in an incubating shaker overnight at 30°C. Cultures were streaked for individual colonies that were then tested for their inability to support plaque formation by KS10. PCR using KS10-specific primers further confirmed the presence of a KS10 prophage. In an attempt to determine the integration sites of KS10 in the three isolated lysogens (as well as strain C5424 and the seven *B. cenocepacia *clinical isolates), PCR using arbitrary primers (ARB6 and ARB2) and a specific KS10 primer were used, and, in addition, the APA gene Gold genome walker kit (Bio S&T, Montreal) was used according to the manufacturer's directions.

## Authors' contributions

ADG, KHL, and KDS carried out the phage genome isolation and sequencing, ADG and KHL participated in the sequence alignment and annotation, and ADG drafted the manuscript. PS carried out additional sequence alignments and generated alignment figures. SS and DSW performed advanced bioinformatic analysis of the predicted genes and proteins. JJD conceived of the study, participated in its design and coordination, and edited the final drafts of the manuscript. All authors read and approved the final manuscript.

## Supplementary Material

Additional file 1 Additional properties of KS10 proteins gp1-gp49. The data provided represent advanced bioinformatic analyses of the predicted proteins encoded on the bacteriophage KS10 genome. Click here for file
