# Supplementary material for: Genomic sequence and activity of KS10, a transposable phage of the Burkholderia cepacia complex
Source: BMC Genomics. 2008 Dec 18;9:615. doi: 10.1186/1471-2164-9-615 (PMC2628397; doi:10.1186/1471-2164-9-615)
Supplement: Additional file 1 — Additional properties of KS10 proteins gp1-gp49. The data provided represent advanced bioinformatic analyses of the predicted proteins encoded on the bacteriophage KS10 genome. [file 1471-2164-9-615-S1.doc]

**Additional File 1.** Additional properties of KS10 proteins gp1-gp49

| **GP#** | **Folding rate** | **SP** | **TM helix** | **TM Barrel** | **Leucine Zipper** | **MW (Da)** | **Cysteine (%)** | | **Theoretical pI** | **Negatively charged residues (Asp + Glu)** | **Positively charged residues**  **(Arg + Lys)** | **Protein**  **stability**  **(II)** | **AA Frequency** | |
| --- | --- | --- | --- | --- | --- | --- | --- | --- | --- | --- | --- | --- | --- | --- |
| 1 | 28.2/sec | None | None | None | None | 45441 | | 0.7 | 6.35 | 45 | 43 | Unstable (42.71) | A-10.49 C-0.73 D-5.85 E-5.12 F-1.71 G-7.56 H-1.46 I-2.20 K-2.68 L-7.56 | M-3.17 N-3.41 P-6.83 Q-4.39 R-7.80 S-5.37 T-6.34 V-10.49 W-3.41 Y-3.41 |
| 2 | -1.9/sec | None | None | None | None | 9099 | | 1.2 | 4.5 | 12 | 6 | Unstable (45.32) | A-13.95 C-1.16 D-9.30 E-4.65 F-1.16 G-4.65 H-2.33 I-4.65 K-2.33  L-18.60 | M-3.49 N-1.16 P-4.65 Q-3.49 R-4.65 S-8.14 T-6.98 V-4.65 W-0.00 Y-0.00 |
| 3 | 5.07/sec | None | None | None | None | 56387 | | 0.8 | 4.64 | 74 | 47 | Unstable (41.13) | A-14.68 C-0.78 D-7.63 E-6.85 F-4.11 G-6.07 H-0.98 I-5.09 K-2.35 L-6.85 | M-3.91 N-2.94 P-4.70 Q-5.09 R-6.85 S-5.28 T-3.72 V-7.63 W-2.35 Y-2.15 |
| 4 | -6.53/sec | None | None | None | None | 61531 | | 1.7 | 6.8 | 72 | 70 | Unstable (42.15) | A-7.81 C-1.67 D-7.62 E-5.76 F-4.83 G-5.76 H-3.16 I-5.58 K-6.13 L-8.74 | M-3.35 N-3.53 P-5.20 Q-4.46 R-6.88 S-5.02 T-2.97 V-7.06 W-2.04 Y-2.42 |

*(continued)*

Table 2 - *Continued*

| **GP #** | **Folding rate** | **SP** | **TM helix** | **TM barrel** | **Leucine zipper** | **MW (Da)** | **Cysteine (%)** | **Theoretical pI** | **Negatively charged residues (Asp + Glu)** | **Positively charged residues (Arg + Lys)** | **Protein stability (II)** | **AA Frequency** | |
| --- | --- | --- | --- | --- | --- | --- | --- | --- | --- | --- | --- | --- | --- |
| 5 | -6.53/sec | None | None | None | None | 61531 | 1.7 | 6.8 | 72 | 70 | Unstable (42.15) | A-7.81 C-1.67 D-7.62 E-5.76 F-4.83 G-5.76 H-3.16  I-5.58 K-6.13  L- 8.74 | M-3.35 N-3.53 P-5.20 Q-4.46 R-6.88 S-5.02 T-2.97 V-7.06 W-2.04 Y-2.42 |
| 6 | -9.43/sec | None | None | None | None | 18826 | 1.2 | 9.37 | 19 | 25 | Unstable (43.98) | A-11.52 C-1.21 D-6.06 E-5.45 F-3.64 G-2.42 H-1.21 I-3.64 K-8.48  L-7.88 | M-4.24 N-4.85 P-2.42 Q-7.27 R-6.67 S-7.27 T-4.85 V-6.67 W-1.21 Y-3.03 |
| 7 | 26.1/sec | None | None | None | 1 (39-70) | 10292 | 3 | 6.06 | 13 | 12 | Stable (7.65) | A-8.33 C-0.00 D-8.33 E-5.21 F-1.04  G-14.58 H-2.08 I-4.17 K-6.25  L-12.50 | M-4.17 N-4.17 P-0.00 Q-6.25 R-6.25 S-1.04 T-7.29 V-7.29 W-0.00 Y-1.04 |
| 8 | -33.8/sec | None | None | None | None | 8448 | 0.6 | 5.38 | 12 | 10 | Unstable (83.37) | A-13.51 C-8.11  D-1.35  E-14.86 F-2.70 G-6.76 H-1.35 I-4.05  K-0.00 L-2.70 | M-4.05 N-1.35 P-5.41 Q-9.46  R-13.51 S-0.00 T-4.05 V-4.05 W-0.00 Y-2.70 |

*(continued)*

Table 2 - *Continued*

| **GP #** | **Folding rate** | **SP** | **TM helix** | **TM barrel** | **Leucine zipper** | **MW (Da)** | **Cysteine (%)** | **Theoretical pI** | **Negatively charged residues (Asp + Glu)** | **Positively charged residues (Arg + Lys)** | **Protein stability (II)** | **AA Frequency** | |
| --- | --- | --- | --- | --- | --- | --- | --- | --- | --- | --- | --- | --- | --- |
| 9 | -7.79/sec | 1-45 | None | None | None | 24501 | 1.8 | 9.59 | 17 | 26 | Unstable (44.50) | A-14.35 C-1.79 D-4.93 E-2.69 F-1.35 G-7.62 H-1.79 I-5.38 K-3.14  L-10.31 | M-1.79 N-3.59 P-6.28 Q-4.48 R-8.52 S-5.38 T-3.59 V-5.83 W-2.69 Y-4.48 |
| 10 | -6.76/sec | None | 20-37 | None | None | 14706 | 1.5 | 6.9 | 12 | 12 | Unstable (47.80) | A-12.03 C-1.50 D-3.76 E-5.26 F-3.76 G-5.26 H-3.01 I-6.02 K-3.01  L-15.04 | M-3.01 N-0.00 P-5.26 Q-1.50 R-6.02 S-6.77 T-3.76 V-9.77 W-2.26 Y-3.01 |
| 11 | 4.33/sec | None | None | None | None | 22504 | 3 | 5.95 | 26 | 25 | Unstable (57.76) | A-9.90 C-2.97 D-6.44 E-6.44 F-2.48 G-3.96 H-0.50 I-4.95 K-5.94 L-8.91 | M-3.47 N-4.95 P-5.45 Q-6.93 R-6.44 S-10.40 T-4.46 V-2.48 W-1.49 Y-1.49 |
| 12 | -10.9/sec | 1-42 | None | None | None | 19638 | 0.6 | 7.91 | 18 | 19 | Stable (22.40) | A-14.69 C-0.56  D-2.82 E-7.34 F-3.39 G-5.08 H-1.13 I-3.95 K-3.39 L-8.47 | M-5.08 N-5.08 P-2.26 Q-3.39 R-7.34 S-6.78 T-6.78 V-7.91 W-3.39 Y-1.13 |

*(continued)*

| Table 2 - *Continued* | | | | | | | | | | | | | |
| --- | --- | --- | --- | --- | --- | --- | --- | --- | --- | --- | --- | --- | --- |
| **GP #** | **Folding rate** | **SP** | **TM helix** | **TM barrel** | **Leucine zipper** | **MW (Da)** | **Cysteine (%)** | **Theoretical pI** | **Negatively charged residues (Asp + Glu)** | **Positively charged residues (Arg + Lys)** | **Protein stability (II)** | **AA Frequency** | |
| 13 | 6.01/sec | 1-25 | None | None | None | 16306 | 0 | 9.45 | 13 | 16 | Stable (19.54) | A-13.46 C-0.00 D-4.49 E-3.85 F-2.56 G-10.26 H-1.92 I-3.21 K-3.85 L-3.85 | M-3.21 N-4.49 P-3.21 Q-5.77 R-6.41 S-7.69 T-8.33 V-11.54 W-0.00 Y-1.92 |
| 14 | 12.6/sec | None | None | None | None | 6998 | 0 | 9.57 | 8 | 12 | Stable (4.37) | A-9.52 C-0.00 D-4.76 E-7.94 F-0.00  G-12.70 H-3.17 I-3.17  K-12.70 L-4.76 | M-3.17 N-1.59 P-1.59 Q-4.76 R-6.35 S-1.59 T-3.17 V-12.70 W-1.59 Y-4.76 |
| 15 | 10.4/sec | 1-25 | None | None | 1 (185-206) | 38672 | 1.1 | 5.99 | 56 | 51 | Stable (39.92) | A-13.14 C-1.14 D-7.14 E-8.86 F-2.86 G-5.71 H-2.29 I-2.86 K-4.29  L-11.43 | M-1.71 N-2.57 P-4.29 Q-2.86 R-10.29 S-5.14 T-4.86 V-7.14 W-0.57 Y-0.86 |
| 16 | 22.9/sec | None | None | None | None | 61199 | 0.6 | 9.31 | 65 | 76 | Unstable (42.97) | A-9.80 C-0.55 D-6.28 E-5.73 F-2.59 G-6.47 H-2.96 I-3.51 K-4.62  L-11.28 | M-1.66 N-2.77 P-4.44 Q-4.62 R-9.43 S-5.55 T-4.99 V-7.21 W-1.48 Y-4.07 |

| Table 2 - *Continued* | | | | | | | | | | | | | |
| --- | --- | --- | --- | --- | --- | --- | --- | --- | --- | --- | --- | --- | --- |
| **GP #** | **Folding rate** | **SP** | **TM helix** | **TM barrel** | **Leucine zipper** | **MW (Da)** | **Cysteine (%)** | **Theoretical pI** | **Negatively charged residues (Asp + Glu)** | **Positively charged residues (Arg + Lys)** | **Protein stability (II)** | **AA Frequency** | |
| 17 | 20.2/sec | None | None | None | None | 36411 | 0.9 | 7.06 | 36 | 36 | Stable (37.01) | A-11.21 C-0.91 D-6.06 E-4.85 F-1.82 G-6.06 H-2.12 I-4.85 K-3.64  L-10.00 | M-3.33 N-4.55 P-3.33 Q-3.94 R-7.27 S-6.67 T-6.97 V-7.88 W-1.52 Y-3.03 |
| 18 | 11.1/sec | None | None | None | None | 6620 | 0 | 10.57 | 4 | 6 | Stable (34.08) | A-19.35 C-0.00 D-3.23 E-3.23 F-4.84 G-6.45 H-3.23 I-3.23 K-0.00 L-4.84 | M-1.61 N-3.23 P-4.84 Q-3.23 R-9.68 S-6.45 T-16.13 V-3.23 W-1.61 Y-1.61 |
| 19 | 20.6/sec | 1-15 | None | None | None | 11775 | 1 | 11.67 | 11 | 23 | Unstable (63.59) | A-11.88 C-0.99 D-5.94 E-4.95 F-0.99 G-4.95 H-0.00 I-0.99 K-0.99 L-14.85 | M-1.98 N-3.96 P-1.98 Q-5.94 R-21.78 S-2.97 T-5.94 V-5.94 W-0.99 Y-1.98 |
| 20 | -10.9/sec | None | None | None | None | 18571 | 0 | 10.14 | 16 | 23 | Unstable (40.22) | A-15.29 C-0.00 D-4.12 E-5.29 F-0.59 G-7.06 H-2.94 I-2.94 K-4.12 L-10.00 | M-4.71 N-1.18 P-8.82 Q-3.53 R-9.41 S-4.71 T-4.71 V-6.47 W-2.35 Y-1.76 |

Table 2 - *Continued*

| **GP #** | **Folding rate** | **SP** | **TM helix** | **TM barrel** | **Leucine zipper** | **MW (Da)** | **Cysteine (%)** | **Theoretical pI** | **Negatively charged residues (Asp + Glu)** | **Positively charged residues (Arg + Lys)** | **Protein stability (II)** | **AA Frequency** | |
| --- | --- | --- | --- | --- | --- | --- | --- | --- | --- | --- | --- | --- | --- |
| 21 | 10.1/sec | None | None | None | None | 23315 | 0.5 | 6.2 | 31 | 30 | Stable (23.24) | A-10.58 C-0.48 D-6.73 E-8.17 F-3.37 G-6.73 H-0.96 I-7.21 K-6.73 L-8.17 | M-1.92 N-2.88 P-1.92 Q-5.77 R-7.69 S-4.33 T-5.29 V-7.69 W-0.96 Y-2.40 |
| 22 | 19.2/sec | None | None | None | None | 14793 | 0.8 | 5.33 | 18 | 15 | Unstable (40.94) | A-8.46 C-0.77 D-8.46 E-5.38 F-4.62 G-8.46 H-1.54 I-5.38 K-1.54 L-10.00 | M-2.31 N-2.31 P-7.69 Q-4.62 R-10.00 S-2.31 T-3.08 V-6.92 W-1.54 Y-4.62 |
| 23 | 10.6/sec | None | None | None | None | 9252 | 0 | 9.7 | 8 | 12 | Stable (25.76) | A-21.11 C-0.00 D-4.44 E-4.44 F-3.33 G-7.78 H-0.00 I-4.44  K-10.00 L-7.78 | M-1.11 N-5.56 P-2.22 Q-6.67 R-3.33 S-5.56 T-4.44 V-7.78 W-0.00 Y-0.00 |
| 24 | 7.19/sec | 1-19 | None | None | None | 30057 | 4 | 4.76 | 40 | 23 | Unstable (52.80) | A-14.08 C-3.97 D-6.14 E-8.30 F-2.89 G-5.42 H-2.53 I-3.25 K-1.81 L-7.58 | M-2.17 N-2.89 P-6.14 Q-4.33 R-6.50 S-7.22 T-4.69 V-6.86 W-1.08 Y-2.17 |

*Table 2 -* Continued

| **GP #** | **Folding rate** | **SP** | **TM helix** | **TM barrel** | **Leucine zipper** | **MW (Da)** | **Cysteine (%)** | **Theoretical pI** | **Negatively charged residues (Asp + Glu)** | **Positively charged residues (Arg + Lys)** | **Protein stability (II)** | **AA Frequency** | |
| --- | --- | --- | --- | --- | --- | --- | --- | --- | --- | --- | --- | --- | --- |
| 25 | 11/sec | None | None | None | None | 11633 | 0.9 | 5.73 | 14 | 11 | Stable (21.06) | A-15.09 C-0.94 D-6.60 E-6.60 F-3.77 G-7.55 H-3.77 I-2.83 K-2.83 L-6.60 | M-2.83 N-1.89 P-2.83 Q-2.83 R-7.55 S-3.77 T-6.60  V-10.38 W-1.89 Y-2.83 |
| 26 | 0.852/sec | None | None | None | None | 16301 | 0.7 | 9.86 | 15 | 22 | Unstable (44.54) | A-18.49 C-0.68 D-4.79 E-5.48 F-1.37 G-3.42 H-2.05 I-1.37 K-6.85  L-13.01 | M-4.11 N-1.37 P-3.42 Q-6.16 R-8.22 S-6.16 T-4.79 V-3.42  W-3.42 Y-1.37 |
| 27 | 3.04/sec | None | None | None | None | 14884 | 0.8 | 6.92 | 16 | 16 | Stable (35.77) | A-9.09 C-0.76 D-4.55 E-7.58 F-3.79 G-6.82 H-3.03 I-6.06 K-2.27  L-10.61 | M-3.79 N-1.52 P-6.82 Q-5.30 R-9.85 S-2.27 T-4.55 V-9.09 W-0.76 Y-1.52 |
| 28 | 3.47/sec | None | None | None | None | 43542 | 0.2 | 5.27 | 54 | 43 | Unstable (40.30) | A-16.00 C-0.25 D-6.50 E-7.00 F-5.25 G-8.00 H-1.75 I-4.25 K-3.00 L-5.00 | M-1.75 N-3.00 P-4.25 Q-5.50 R-7.75 S-5.50 T-4.75 V-7.25 W-2.50 Y-0.75 |

*(continued)*

Table 2 - *Continued*

| **GP #** | **Folding rate** | **SP** | **TM helix** | **TM barrel** | **Leucine zipper** | **MW (Da)** | **Cysteine (%)** | **Theoretical pI** | **Negatively charged residues (Asp + Glu)** | **Positively charged residues (Arg + Lys)** | **Protein stability (II)** | **AA Frequency** | |
| --- | --- | --- | --- | --- | --- | --- | --- | --- | --- | --- | --- | --- | --- |
| 29 | 7.84/sec | None | None | None | None | 7011 | 0 | 4.99 | 8 | 6 | Stable (36.11) | A-18.18 C-0.00 D-9.09 E-3.03 F-4.55 G-6.06 H-1.52 I-4.55 K-6.06 L-3.03 | M-3.03 N-1.52 P-4.55 Q-7.58 R-3.03 S-7.58 T-6.06 V-7.58 W-1.52 Y-1.52 |
| 30 | 0.0612/sec | None | None | None | None | 11536 | 0 | 5.17 | 10 | 12 | Stable (4.99) | A-22.03 C-0.00 D-7.63 E-2.54 F-0.00  G-14.41 H-0.85 I-5.93 K-2.54 L-10.17 | M-0.85 N-1.69 P-3.39 Q-0.85 R-5.93 S-3.39 T-5.93 V-9.32 W-0.00 Y-2.54 |
| 31 | 10.6/sec | None | None | None | None | 34304 | 0.3 | 5.26 | 38 | 32 | Stable (35.13) | A-11.75 C-0.32 D-6.67 E-5.40 F-3.81 G-7.30 H-0.95 I-5.08 K-3.17 L-8.57 | M-1.59 N-4.44 P-6.98 Q-5.71 R-6.98 S-3.49 T-6.35 V-8.57 W-0.63 Y-2.22 |
| 32 | 8.27/sec | None | None | None | None | 13026 | 0 | 9.39 | 15 | 18 | Unstable (40.38) | A-15.50 C-0.00 D-4.65 E-6.98 F-0.78  G-12.40 H-2.33 I-0.78 K-6.98 L-3.10 | M-1.55 N-0.78 P-6.20 Q-3.10 R-6.98 S-11.63 T-7.75 V-7.75 W-0.00 Y-0.78 |

*(continued)*

Table 2 - *Continued*

| **GP #** | **Folding rate** | **SP** | **TM helix** | **TM barrel** | **Leucine zipper** | **MW (Da)** | **Cysteine (%)** | **Theoretical pI** | **Negatively charged residues (Asp + Glu)** | **Positively charged residues (Arg + Lys)** | **Protein stability (II)** | **AA Frequency** | |
| --- | --- | --- | --- | --- | --- | --- | --- | --- | --- | --- | --- | --- | --- |
| 33 | 7.69/sec | None | None | None | None | 19046 | 3 | 4.74 | 28 | 21 | Unstable (54.28) | A-8.38 C-2.99 D-8.98 E-7.78 F-1.20 G-4.79 H-0.00 I-0.60 K-3.59  L-12.57 | M-2.40 N-2.40 P-5.39 Q-5.39 R-8.98 S-6.59 T-7.19 V-4.19 W-2.40 Y-4.19 |
| 34 | 9.79/sec | None | None | None | None | 15859 | 0 | 6.04 | 18 | 16 | Unstable (43.65) | A-9.09 C-0.00 D-9.79 E-2.80 F-2.10  G-11.19 H-2.80 I-6.29 K-1.40 L-6.99 | M-4.20 N-2.80 P-1.40 Q-5.59 R-9.79 S-6.29 T-3.50 V-8.39 W-1.40 Y-4.20 |
| 35 | 1.65/sec | None | None | None | None | 22377 | 1 | 5.09 | 28 | 22 | Stable (39.36) | A-10.50 C-1.00 D-5.00 E-9.00 F-4.00 G-6.50 H-1.00 I-3.50 K-5.00 L-6.50 | M-4.00 N-1.00 P-5.50 Q-3.50 R-6.00 S-6.50 T-7.00 V-8.50 W-3.00 Y-3.00 |
| 36 | -13.6/sec | None | None | None | None | 9657 | 0 | 4.86 | 12 | 9 | Stable (36.19) | A-13.04 C-0.00 D-9.78 E-3.26 F-1.09 G-8.70 H-1.09 I-3.26 K-3.26 L-5.43 | M-4.35 N-2.17 P-6.52 Q-3.26 R-6.52 S-4.35 T-9.78  V-11.96 W-0.00 Y-2.17 |

*(continued)*

Table 2 - *Continued*

| **GP #** | **Folding rate** | **SP** | **TM helix** | **TM barrel** | **Leucine zipper** | **MW (Da)** | **Cysteine (%)** | **Theoretical pI** | **Negatively charged residues (Asp + Glu)** | **Positively charged residues (Arg + Lys)** | **Protein stability (II)** | **AA Frequency** | |
| --- | --- | --- | --- | --- | --- | --- | --- | --- | --- | --- | --- | --- | --- |
| 37 | -11.3/sec | None | None | None | None | 53071 | 0.8 | 5.09 | 51 | 38 | Stable (30.02) | A-11.59 C-0.81 D-5.28 E-5.08 F-2.44 G-8.13 H-1.42 I-6.50 K-3.46 L-7.72 | M-3.86 N-5.28 P-4.88 Q-2.64 R-4.27 S-4.88 T-8.54 V-8.33 W-1.83 Y-3.05 |
| 38 | 4.27/sec | None | None | None | None | 13661 | 2.4 | 5.1 | 17 | 14 | Unstable (40.74) | A-6.50 C-2.44 D-8.13 E-5.69 F-6.50 G-8.13 H-0.81 I-4.07 K-4.88 L-4.88 | M-4.07 N-1.63 P-4.07 Q-4.88 R-6.50 S-7.32 T-8.94 V-8.13 W-1.63 Y-0.81 |
| 39 | 12.9/sec | 1-21 | None |  | None | 20101 | 1.6 | 4.82 | 30 | 21 | Stable (24.85) | A-12.50 C-1.63 D-9.78 E-6.52 F-2.17 G-7.07 H-1.63 I-3.26 K-2.72 L-11.96 | M-3.26 N-1.09 P-2.17 Q-4.89 R-8.70 S-5.98 T-5.43 V-7.07 W-0.00 Y-2.17 |
| 40 | 3.78/sec | None | None | None | None | 82943 | 0 | 9.85 | 66 | 86 | Stable (32.81) | A-17.51 C-0.00 D-5.18 E-2.96 F-1.97 G-12.33 H-0.25 I-3.70 K-4.19 L-8.75 | M-3.58 N-4.32 P-3.45 Q-4.56 R-6.41 S-6.17 T-6.66 V-6.04 W-0.62 Y-1.36 |

(continued)

| **GP #** | **Folding rate** | **SP** | **TM helix** | **TM barrel** | **Leucine zipper** | **MW (Da)** | **Cysteine (%)** | **Theoretical pI** | **Negatively charged residues (Asp + Glu)** | **Positively charged residues (Arg + Lys)** | **Protein stability (II)** | **AA Frequency** | |
| --- | --- | --- | --- | --- | --- | --- | --- | --- | --- | --- | --- | --- | --- |
| 41 | 1.13/sec | None | None | None | None | 49389 | 0.2 | 4.78 | 52 | 35 | Stable (33.74) | A-13.82 C-0.22 D-7.46 E-3.95 F-3.07 G-5.70 H-1.32 I-5.04 K-2.19  L-10.09 | M-1.75 N-3.29 P-5.04 Q-4.17 R-5.48 S-8.33 T-6.80 V-7.02 W-2.19 Y-3.07 |
| 42 | 13.9/sec | None | None | None | None | 42538 | 1.3 | 8.8 | 51 | 56 | Stable (29.00) | A-7.79 C-1.30 D-7.53 E-5.71 F-2.34 G-8.83 H-2.34 I-5.97 K-6.75 L-6.75 | M-1.82 N-3.12 P-3.64 Q-2.60 R-7.79 S-5.45 T-5.71  V-10.65 W-1.56 Y-2.34 |
| 43 | 0.365/sec | None | None | None | None | 18867 | 0.6 | 5.46 | 25 | 19 | Stable  (32.33) | A-7.51 C-0.58 D-9.25 E-5.20 F-2.89  G-10.40 H-3.47 I-4.62 K-3.47 L-7.51 | M-2.31 N-5.78 P-2.31 Q-4.05 R-7.51 S-4.62 T-6.36 V-10.40 W-0.58 Y-1.16 |
| 44 | 1.84/sec | 1-25 | None | None | None | 21648 | 2.1 | 4.84 | 28 | 19 | Stable (34.62) | A-12.44 C-2.07 D-9.84 E-4.66 F-3.11 G-4.15 H-2.07 I-4.15 K-1.04 L-8.81 | M-1.55 N-2.59 P-3.63 Q-2.07 R-8.81 S-7.77 T-8.29 V-6.74 W-4.66 Y-1.55 |

Table 2 - *Continued*

*(continued)*

*Table 2 -* Continued

| **GP #** | **Folding rate** | **SP** | **TM helix** | **TM barrel** | **Leucine zipper** | **MW (Da)** | **Cysteine (%)** | **Theoretical pI** | **Negatively charged residues (Asp + Glu)** | **Positively charged residues (Arg + Lys)** | **Protein stability (II)** | **AA Frequency** | |
| --- | --- | --- | --- | --- | --- | --- | --- | --- | --- | --- | --- | --- | --- |
| 45 | 1.42/sec | 1-16 | None | None | None | 39279 | 0.3 | 4.83 | 43 | 30 | Stable (28.22) | A-14.21 C-0.27 D-6.70 E-4.83 F-3.22 G-8.85 H-1.07 I-5.09 K-2.14 L-8.58 | M-0.80 N-2.14 P-5.90 Q-4.29 R-5.90 S-5.36 T-7.24  V-10.19 W-0.80 Y-2.41 |
| 46 | -13.6/sec | 1-34 | None | None | None | 22676 | 3 | 6.14 | 29 | 27 | Unstable (49.01) | A-10.61 C-3.03 D-7.58 E-7.07 F-3.54 G-6.57 H-2.02 I-3.54 K-1.52  L-11.11 | M-2.02 N-2.53 P-5.05 Q-2.02 R-12.1 S-3.54 T-2.53 V-7.58  W-3.03 Y-3.03 |
| 47 | 7.72/sec | None | None | None | None | 34639 | 0.6 | 5.46 | 39 | 30 | Stable(26.63) | A-12.62 C-0.62 D-8.31 E-3.69 F-2.15 G-12.00 H-2.77 I-4.62 K-2.46 L-6.77 | M-2.46 N-5.23 P-3.69 Q-2.46 R-6.77 S-6.77 T-4.62 V-6.77 W-1.85 Y-3.38 |
| 48 | 23.7/sec | None | None | None | None | 80473 | 0.9 | 4.87 | 90 | 66 | Stable (31.46) | A-11.46 C-0.94 D-7.82 E-4.31 F-3.64 G-9.30 H-1.08 I-3.64 K-2.56 L-7.68 | M-1.75 N-4.31 P-5.26 Q-3.77 R-6.33 S-5.93 T-5.80 V-8.63 W-1.89 Y-3.91 |

| **GP #** | **Folding rate** | **SP** | **TM helix** | **TM barrel** | **Leucine zipper** | **MW (Da)** | **Cysteine (%)** | **Theoretical pI** | **Negatively charged residues (Asp + Glu)** | **Positively charged residues (Arg + Lys)** | **Protein stability (II)** | **AA Frequency** | |
| --- | --- | --- | --- | --- | --- | --- | --- | --- | --- | --- | --- | --- | --- |
| 49 | 11.5/sec | None | None | None | None | 26957 | 1.2 | 5.2 | 26 | 19 | Stable (28.72) | A-9.84 C-1.18 D-5.51 E-4.72 F-5.51  G-11.42 H-1.97 I-5.91 K-2.36 L-8.27 | M-1.97 N-3.94 P-3.94 Q-1.97 R-5.12 S-9.45 T-6.69 V-6.69 W-0.79 Y-2.76 |

Table 2 - *Continued*

Abbreviations: GP-gene product; SP-signal peptide; TM- transmembrane; MW- molecular weight; pI- isoelectric point; AA- amino acid
